# Right Atrial Thrombus in a Patient With COVID-19

**DOI:** 10.7759/cureus.9441

**Published:** 2020-07-28

**Authors:** Vittorio R Terrigno, Jian Liang Tan, Devinder Singh, Sajjad A Sabir

**Affiliations:** 1 Internal Medicine, Cooper University Hospital, Camden, USA; 2 Cardiology, Cooper University Hospital, Camden, USA

**Keywords:** covid 19, right atrial thrombus, thrombolytics, anticoagulation

## Abstract

Coronavirus disease 2019 (COVID-19) is a worldwide pandemic. Evidence suggests a strong association between COVID-19 and pro-thrombotic states. We report our experience in managing a patient with COVID-19 complicated by a right atrial thrombus. We highlight the successful use of half-dose anticoagulation in the treatment of right atrial thrombus in a patient with COVID-19. To our knowledge, this is a first reported case of right atrial thrombus in a COVID-19 patient who was treated successfully with half-dose anticoagulation.

## Introduction

Coronavirus disease 2019 (COVID-19) is a dangerous infectious disease that can affect multiple organ systems. While no definitive treatment has been established, it is apparent that early diagnosis is essential for the initiation of supportive measures and treatment with the end goal of preventing rapid respiratory collapse. Hypercoagulable states as well as the many complications that can be associated with an increased state of coagulation have been strongly associated with this disease. It is important that the medical community continues to discuss and examine the appropriate preventative and therapeutic interventions to combat this specific aspect of this disease course. 

## Case presentation

**Figure 1:**
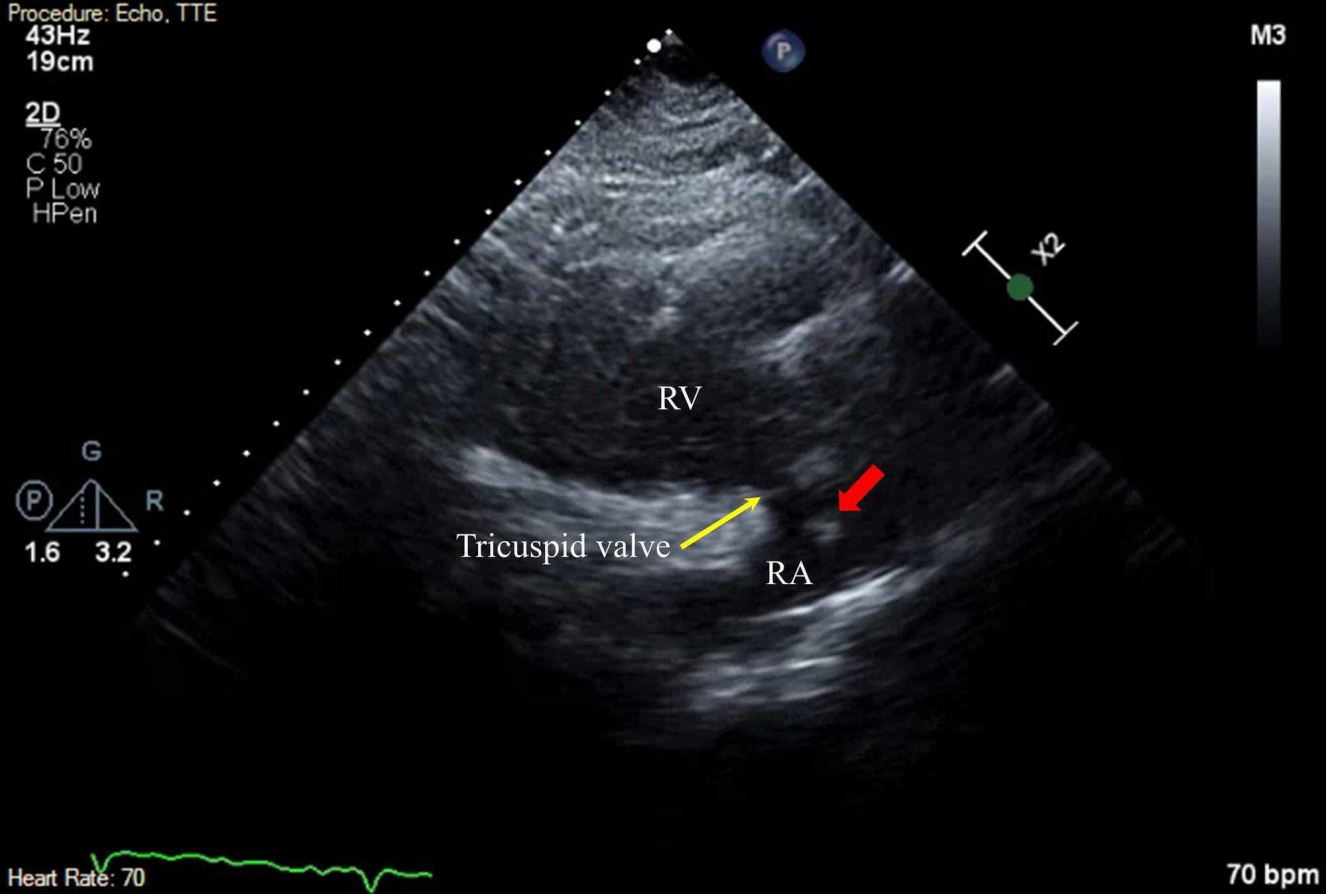
Right Atrial Thrombus Two-dimensional TTE finding. Parasternal long-axis RV inflow tract view illustrating a mobile echo dense mass (red arrow) in the RA. RA, right atrium; RV, right ventricle; TTE, transthoracic echocardiogram

**Figure 2:**
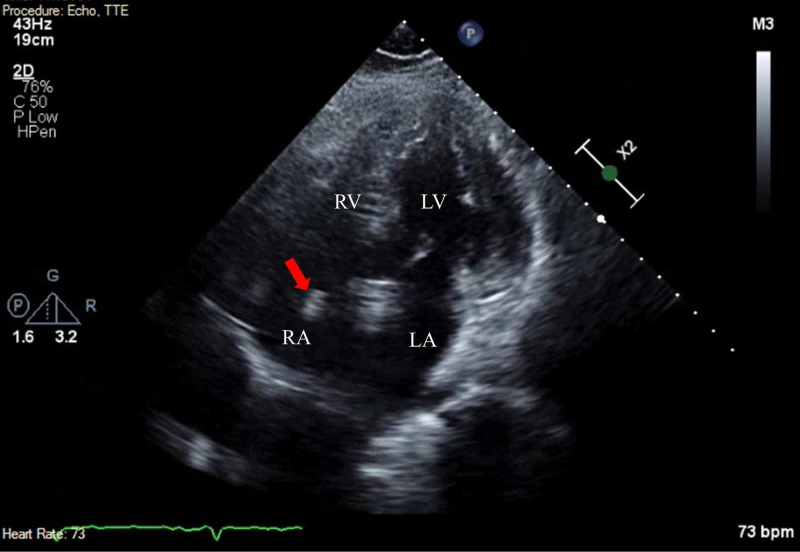
Right Atrial Thrombus: Apical Four-Chamber View Two-dimensional TTE finding. Apical four-chamber view illustrating a mobile echo dense mass (red arrow) in the RA. LA, left atrium; LV, left ventricle; RA, right atrium; RV, right ventricle; TTE, transthoracic echocardiography

A 76-year-old woman with asthma and hypertension presented to the emergency room with a one-week history of dyspnea. She had an exposure to an individual who tested positive for severe acute respiratory syndrome coronavirus 2 (SARS-CoV-2). On examination, she was able to speak in full sentences. Her oxygenation saturation was 66% on room air, which improved to 94% with a non-rebreather facemask. Wheezing and diminished breath sounds were present on lung auscultation. Her respiratory status declined with an increased work of breathing within 30 minutes of her presentation, which prompted endotracheal intubation. Her intubation course was complicated due to significant laryngeal edema and a brief episode of pulseless ventricular tachycardia with return of spontaneous circulation after three cycles of chest compressions. She required intravenous (IV) norepinephrine to achieve a mean arterial pressure of 65 mm Hg. Laboratory tests demonstrated lymphopenia and abnormal biomarkers (creatinine of 1.7 mg/dL, procalcitonin of 0.33 ng/mL, C-reactive protein of 26 mg/dL, D-dimer of 6.8 ug/mL, lactate dehydrogenase of 1,132 U/L, ferritin of 1,102 ng/mL, and lactate of 2.2 mmol/L). Her initial high-sensitivity troponin was 472 ng/L and peaked at 606 mg/L. Post-cardiac arrest transthoracic echocardiogram (TTE) revealed reduced biventricular systolic function and a mobile echo dense mass in the right atrium (RA) (Figures 1, 2 and Videos 1, 2).

**Video 1:** Parasternal Long Axis View:- Mobile Echogenic Mass in the Right Atrium Transthoracic echocardiogram in parasternal long-axis right ventricular inflow tract view showing a mobile echogenic mass in the right atrium prolapsing through the tricuspid valve inlet.

**Video 2:** Apical Five-Chamber View: Mobile Echogenic Mass in the Right Atrium Transthoracic echocardiogram in apical five-chamber view showing a mobile echogenic mass in the right atrium and global hypokinesis of the left and right ventricles.

An agitated saline study did not show evidence of right to left shunting (Video 3). She was positive for SARS-CoV-2 based on a real-time reverse transcription polymerase chain reaction (rRT-PCR).

**Video 3:** Agitated Saline Study An agitated saline study showing no evidence of right to left shunting.

Given her hemodynamic profile (increased dose of norepinephrine), a multidisciplinary team discussed various therapeutic approaches (systemic thrombolysis and surgical embolectomy) to manage the clot in the RA. A decision was made to administer 50 mg of alteplase as a bolus over 2 minutes followed by therapeutic anticoagulation with IV heparin. This decision was made due to the patient’s need for proning due to acute respiratory distress syndrome and also her need for close monitoring for potential bleeding. Due to these variables, which many patients with COVID-19 face, half-dose alteplase therapy was given instead of the full dose. A repeat TTE was performed on hospital day 4, which demonstrated resolution of the clot (Video 4).

**Video 4:** TTE demonstrating RA Clot Resolution TTE in apical four-chamber view at day 4 post-tissue plasminogen activator administration and while on continuous heparin infusion showed absence of RA clot and improvement in biventricular systolic function. TTE, transthoracic echocardiography; RA, right atrium

She was eventually taken off of IV norepinephrine on hospital day 9 and showed some clinical signs of improvement. However, despite receiving hydroxychloroquine, azithromycin, and lopinavir/ritonavir, her respiratory status once again worsened. Her family decided to proceed with a compassionate extubation due to worsening of her COVID-19 illness and she expired on hospital day 13. Due to hospital protocol during the COVID-19 pandemic and the family’s wish, autopsy was not performed.

## Discussion

In a retrospective single center study of 81 COVID-19 ICU patients from China, 25% (20/81) of the patients developed venous thromboembolism (VTE). These patients had an elevated D-dimer, similar to our patient. None of the patients had received VTE prophylaxis [1]. In another three-center retrospective study of 184 COVID-19 ICU patients from the Netherlands, the authors reported 31% incidence of thrombotic complications. All patients received standard thromboprophylaxis. Of note, the diagnostic testing was only carried out in patients with a suspicion of thrombotic complications [2]. Therefore, the true incidence is likely higher.

Our case supports the need to think of initiating therapeutic anticoagulation early in the course of critically ill patients with COVID-19. In a publication by an international collaborative of clinicians and investigators, a majority of the panel members recommended prophylactic dose anticoagulation, but a minority supported considering even intermediate to therapeutic dose anticoagulation [3]. In addition to this, our case also highlights the use of half-dose anticoagulation therapy to treat a right atrial thrombus. Patients with COVID-19 may need prone positioning, which makes monitoring for bleeding difficult and frequent neurological checks difficult. In addition to this, patients with other bleeding risks may not be able to receive full-dose anticoagulation. To our knowledge, this is the first reported case of right atrial thrombus in a COVID-19 patient that resolved with a half dose of thrombolytic therapy.

## Conclusions

Our case report highlights the continued need for the consideration of early anticoagulation in patients with COVID-19. Numerous studies have shown a direct correlation between hypercoagulable states and COVID-19 infection. We highlight the ability to treat a right atrial thrombus in a patient with COVID-19 with half-dose anticoagulation. This may be beneficial in patients with COVID-19 who require prone positioning and frequent neurological checks as well as for COVID-19 patients who are at higher risk when receiving thrombolytic therapy.
